# Parthenolide inhibits the growth of non-small cell lung cancer by targeting epidermal growth factor receptor

**DOI:** 10.1186/s12935-020-01658-1

**Published:** 2020-11-23

**Authors:** Xiaoling Li, Riming Huang, Mingyue Li, Zheng Zhu, Zhiyan Chen, Liao Cui, Hui Luo, Lianxiang Luo

**Affiliations:** 1 Experimental Animal Center, Guangdong Medical University, Zhanjiang, 524023 Guangdong China; 2 Guangdong Provincial Key Laboratory of Food Quality and Safety, College of Food Science, South China Agricultural University, Guangzhou, 510642 Guangdong China; 3 Department of Pathology and Laboratory Medicine, Perelman School of Medicine, University of Pennsylvania, Philadelphia, PA USA; 4 Department of Internal Medicine, Division of Hematology/Oncology, University of California Davis, Sacramento, CA 95817 USA; 5 The First Clinical College, Guangdong Medical University, Zhanjiang, 524023 Guangdong China; 6 Guangdong Key Laboratory for Research and Development of Natural Drugs, Guangdong Medical University, Zhanjiang, 524023 Guangdong China; 7 The Marine Biomedical Research Institute, Guangdong Medical University, Zhanjiang, 524023 Guangdong China; 8 The Marine Biomedical Research Institute of Guangdong Zhanjiang, Zhanjiang, 524023 Guangdong China

**Keywords:** EGFR, NSCLC, Parthenolide, In vitro, In vivo

## Abstract

**Background:**

EGFR tyrosine kinase inhibitors (TKIs) have been developed for the treatment of EGFR mutated NSCLC. Parthenolide, a natural product of parthenolide, which belongs to the sesquiterpene lactone family and has a variety of biological and therapeutic activities, including anti-cancer effects. However, its effect on non-small cell lung cancer is little known.

**Methods:**

The CCK8 assay and colony formation assays were used to assess cell viability. Flow cytometry was used to measure the cell apoptosis. In silico molecular docking was used to evaluate the binding of parthenolide to EGFR. Network pharmacology analysis was was used to evaluate the key gene of parthenolide target NSCLC. Western blotting was used to evaluate the key proteins involved apoptosis and EGFR signalling. The effect of parthenolide treatment in vivo was determined by using a xenograft mouse model.

**Results:**

In this study, parthenolide could induce apoptosis and growth inhibition in the EGFR mutated lung cancer cells. Parthenolide also reduces the phosphorylation of EGFR as well as its downstream signaling pathways MAPK/ERK and PI3K/Akt. Molecular docking analysis of EGFR binding site with parthenolide show that the anti-cancer effect of parthenolide against NSCLC is mediated by a strong binding to EGFR. Network pharmacology analysis show parthenolide suppresses NSCLC via inhibition of EGFR expression. In addition, parthenolide inhibits the growth of H1975 xenografts in nude mice, which is associated with the inhibition of the EGFR signaling pathway.

**Conclusions:**

Taken together, these results demonstrate effective inhibition of parthenolide in NSCLC cell growth by targeting EGFR through downregulation of ERK and AKT expression, which could be promisingly used for patients carrying the EGFR mutation.

## Introduction

Lung cancer is the leading cause of cancer-related deaths worldwide, causing about 1.6 million death [1]. Non-small cell lung cancer (NSCLC) accounts for the vast majority of lung cancer cases (approximately 85%), and it is usually diagnosed at an advanced stage with poor prognosis [2, 3]. Conventional treatment strategies for NSCLC includes surgery, chemotherapy, and radiotherapy. In NSCLC cases, about 30% of patients carry the epidermal growth factor receptor (EGFR) mutation [4]. Currently, tyrosine kinase-based inhibitors(TKIs) molecular-targeted therapy has been proved as a good candidate for NSCLC patients with EGFR mutation [5].

The EGFR signaling pathway is critical to the growth and proliferation of Mammalian cells [6]. Overexpression of EGFR activates downstream signaling pathways including PI3K/AKT and MEK/ERK signaling, resulting in aggressive growth and invasive related phenotypes in cells [7], such as tumor cell motility, adhesion, metastasis, as well as angiogenesis. Therefore, blocking the overexpression of EGFR will inhibit the growth of lung cancer [8, 9].

Chinese herbal medicine are important sources of anticancer drug development [10, 11]. Parthenolide, an abundant sesquiterpene, which was first found in the medicinal plant feverfew (*Tanacetum parthenium*) [12]. It has a variety of pharmacological activities and is used to treat a variety of diseases including arthritis, fever and headache. Recently, parthenolide showed an anticancer activity on various tumors including colorectal cancer, melanoma, breast cancer, pancreatic cancer, prostate cancer [13–15]. A growing evidence indicates that parthenolide induces apoptosis by increasing oxidative stress, mitochondrial dysfunction [16], NF-κB activity inhibition [17] as well as JAK-STAT3 signaling inhibition [18]. Although parthenolide suppressed in vivo tumor growth of lung cancer by targeting NF-κB target and inducing ROS [19], the basic oncogenic role of EGFR in NSCLC and its specific pharmacological of parthenolide in NSCLC is little known. In the study, we aimed to further investigate the cytotoxicity of parthenolide to NSCLC, and illustrate its mechanism as EGFR inhibitor as well as identifying it as a promising therapeutic strategy for EGFR mutated NSCLC.

## Materials and methods

### Reagents and antibodies

Parthenolide was purchased from MedChemExpress (cat# HY-N0141) and dissolved in DMSO. The Annexin V-FITC/PI staining kit was was purchased from Thermofisher (cat#88-8005-72). The antibodies for western blot are as followings: primary antibodies against phospho-EGFR (cat#3777), total-EGFR (cat#4267), phospho-ERK (cat#4377), total-ERK (cat#4695), phospho-AKT(T473) (cat#4060), phospho-AKT(T308) (cat#13,038), AKT (cat#9272), cleaved-caspase-3 (cat#9661), PARP (cat#9532), Ki-67(cat#9027), HRP goat anti-rabbit (cat#7074) and-mouse secondary antibodies (cat#5127) and GAPDH (cat#5174) were purchased from CST Cell Signaling Technology.

### Cell culture

The human NSCLC cell lines ( H1975, PC-9, HCC827, H358, H460 and A549) were obtained from the American Type Culture Collection (ATCC), Cells were cultured in DMEM or RPMI1640 medium which supplemented with 10% FBS (Gibco, USA). The human normal lung epithelial BEAS-2B cells were grown in BEBM medium containing 0.01 mg/mL bovine serum albumin, 0.03 mg/mL bovine collagen type I and 0.01 mg/mL fibronectin. All the cell lines were cultured at 37 °C in a humidified environment containing 5% CO2.

### Cell viability assay

Cell viability was determined by cell counting Kit-8 (Dojindo). Briefly, 5000 cells/well were seeded in 96-well plate for 24 h, and treated with increased concentrations of parthenolide for an additional 24 h, then 10 ul CCK8 reagent was added and incubated for 3 h. The absorbance was measured using a microplate reader at 450 nm. The percent of growth is calculated by normalising against the control cells, and the IC_50_ value was calculated using GraphPad Prism7.

### Colony formation assay

Briefly, 1000 cells/well (H1975, PC-9 and HCC827) were seeded in 6-well plate, cells were exposed to various concentration of parthenolide, The culture medium was changed every 3 days until the visible colonies were formed. The colony was washed with cold PBS, fixed with 4% paraformaldehyde for 15 min, then stained with 0.5% crystal violet for 20 min. The colonies were photographed.

### Apoptosis assay

Cells were resuspended in 1 × 100 µl binding buffer, propidium iodide (5 µl) and Annexin-V (5 µl) were added to the solution, mixed well and incubated in dark for 20 min at room temperature. Cells were washed with 1ebinding buffer and centrifuged at 400 g at 4 °C for 5 minutes. Then cell pellets were resuspended and apoptosis assay was performed on a Beckman CytoFLEX FCM. The percentage of apoptotic cells was identified by both of early apoptotic (PI- Annexin V+) and late apoptic (PI + Annexin V+).

### Western blot

Cells were harvested in yysis buffer, and supernatant was centrifuged at 12,000 *g* for 10 min. Extract was quantified with bradford method. Lysate was heated to 100 °C in SDS sample buffer with 50 mM DTT for 5 min. Protein was separated by 10% SDS-PAGE gel and transferred to PVDF membrane. Blots were blocked for 1 h in TBS-T containing 5% non-fat dry milk, incubated with primary antibodies at 4 °C overnight. After washing, blots were incubated at room temperature with secondary antibodies labeled with conjugated to HRP for 1 h. Finally, blots were detected using the LICOR system for enhanced chemiluminescence.

### Molecular docking

Download EGFR (PDB ID:4HJO) from the RSCB PDB database (http://www.rcsb.org/) and save it as an PDB format file. Then it was imported into AutoDock 4 software for pretreatment, including extraction of ligand small molecules, removal of water molecules, hydrogenation and so on. The sdf file of parthenolide3D structure downloaded from PubChem database was optimized, and parthenolide was docked with protein by AutoDock 4. PyMol were employed to generate the docking input files and to analyze the docking results.

### Construction of protein-protein interaction (PPI) network

The 3D structure of parthenolide was obtained by pubchem (https://pubchem.ncbi.nlm.nih.gov/)[20] and imported into pharmmapper server (http://www.lilab-ecust.cn/pharmmapper/) for parthenolide target prediction [21]. The specific setting is Generate Conformers: yes; Maximum Generated Conformations: 300; Select Target Set: Human Protein Target Only; Number of Reserved Matched Targets: 300. A total of 243 predetermined proteins were obtained. A total of 58 proteins with ZValue > 0.8 were screened. Fifty eight target protein genes were introduced into STRING (https://string-db.org/) database [22] and Uniprot database for gene correction[23]. Fifty six target protein genes were successfully transformed into gene abbreviations, of which BRAF1_HUMAN and PTGD2_HUMAN were not transformed successfully. The transformed 56 protein genes were further introduced into STRING. Under the condition that the confidence score score > 0.4 and hiding the unconnected nodes in the network, a co-expression network constructed by 51 qualified protein genes was obtained. The co-expression network was analyzed by STRING, downloaded chart information, and beautified by cytoscape software[24].

### Mouse xenograft assay

The animal experiments involved in this experiment were conducted according to the Animal Ethics Committee of Guangdong Medical University (GDY1902062) and and committee-approved protocols. 2.5 × 10^6^ H1975 cells were resuspended in FBS-free medium and injected subcutaneously in 100 µl volume into the 6-week-old nude mice. The tumor xenografts were allowed to grow to ~ 60 mm^3^, and five mice per group were treated with vehicle (2% DMSO, 40% PEG400, and 2% Tween 80 in normal saline) or parthenolide (20 mg/kg) via intraperitoneal (ip) injection daily for 14 days. Body weight was monitored every day. Tumors were measured every three days using a digital caliper, and tumor volumes (mm^3^) were calculated using the following formula: volume (mm^3^) = length (mm) × width (mm)^2^ × π/6.

### Histology and immunohistochemistry

Tumor tissues were fixed in 10% formalin and embedded in paraffin. The sections were dewaxed in xylene and dehydrated in alcohol. The sections were stained with hematoxylin and eosin (H&E) for histology analysis. For immunohistochemistry, the sections were incubated with 0.01M sodium citrate (pH = 6.0) for antigen retrieval, endogenous peroxidase was blocked by adding 0.3% H_2_O_2_ and incubated for 30 min in a 10% goat serum albumin. The sections were incubated overnight at 4 °C with antibodies to p-ERK, p-AKT, p-EGFR, Ki-67 and cleaved caspase-3 from Cell Signaling Technology. Samples were incubated with a HRP-conjugated anti-rabbit secondary antibody for 1 h, and signal was visualized by incubation with diaminobenzidine. Finally, the sections were counterstained with Mayer’s haematoxylin, dehydrated, cleared in xylene and mounted in permount TM mounting medium.

### Statistical analysis

The results are presented as the mean ± SD. The statistical significance of differences was assessed using the ANOVA and Student’s *t-*test. *P* < 0.05 were considered statistically significant.

## Results

### 1. Parthenolide inhibits cell proliferation and colony formation in EGFR mutated NSCLC cells

**Fig. 1:**
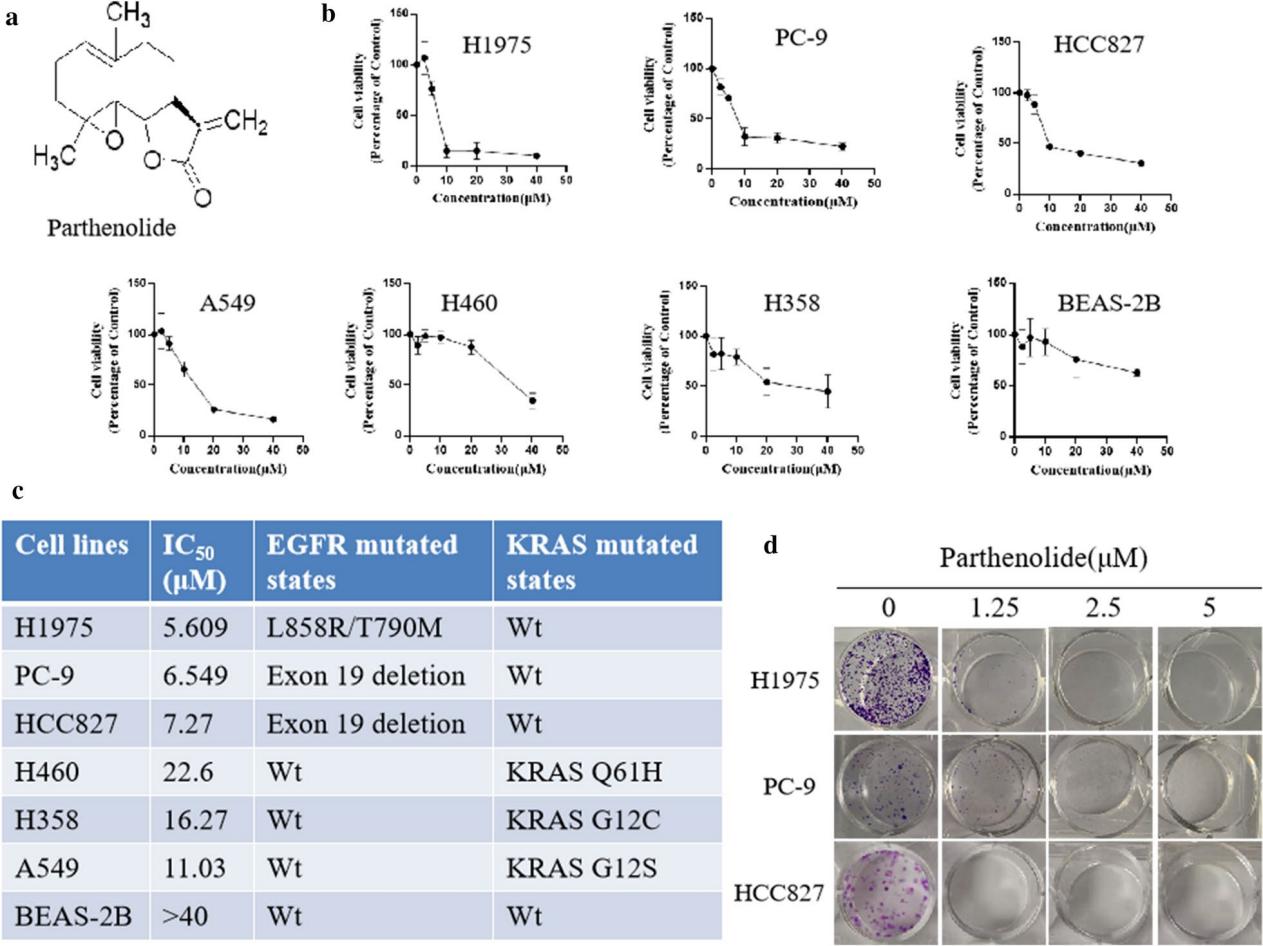
Parthenolide inhibits NSCLC cell growth and colony formation. **a** Chemical structure of parthenolide. **b** The NSCLC and normal cells (H1975, PC-9, HCC827, H460, H358, A549 and BEAS-2B) were treated with various concentrations of parthenolide (0, 2.5, 5, 10, 20 and 40 µM) for 24 h, and cell viability was determined using the CCK8 assay. **c** H1975, PC-9 and HCC827 cells were treated with parthenolide at different concentrations (0, 1.25, 2.5 and 5 µM) for 10 days. Colonies were stained with crystal violet, and representative photomicrographs of crystal violet stained colonies were depicted

The structure of parthenolide was shown in Fig. 1a. Firstly, the cell viability was evaluated after parthenolide treatment. Panels of NSCLC cells were treated with parthenolide at different concentrations (0, 2.5, 5, 10, 20, 40 µM) for 24 h. A dose-dependent viability inhibition of NSCLC cells was observed. As shown in Fig. 1b, parthenolide potently inhibited the growth of several NSCLC cell lines including A549, H460, H358, H1975, HCC827 and PC-9. Interestingly, parthenolide more potently inhibited the growth of EGFR mutated NSCLC cell lines than either EGFR wt NSCLC or normal lung BEAS-2B cells (Fig. 1c). To confirm this finding, we studied the effect of parthenolide on colony formation of EGFR mutant cell lines. According to Cytotoxicity, parthenolide inhibited the colony formation of EGFR mutant cells in a dose-dependent manner (Fig. 1d).

### 2. Parthenolide induces apoptosis in EGFR mutated cell lines

**Fig. 2:**
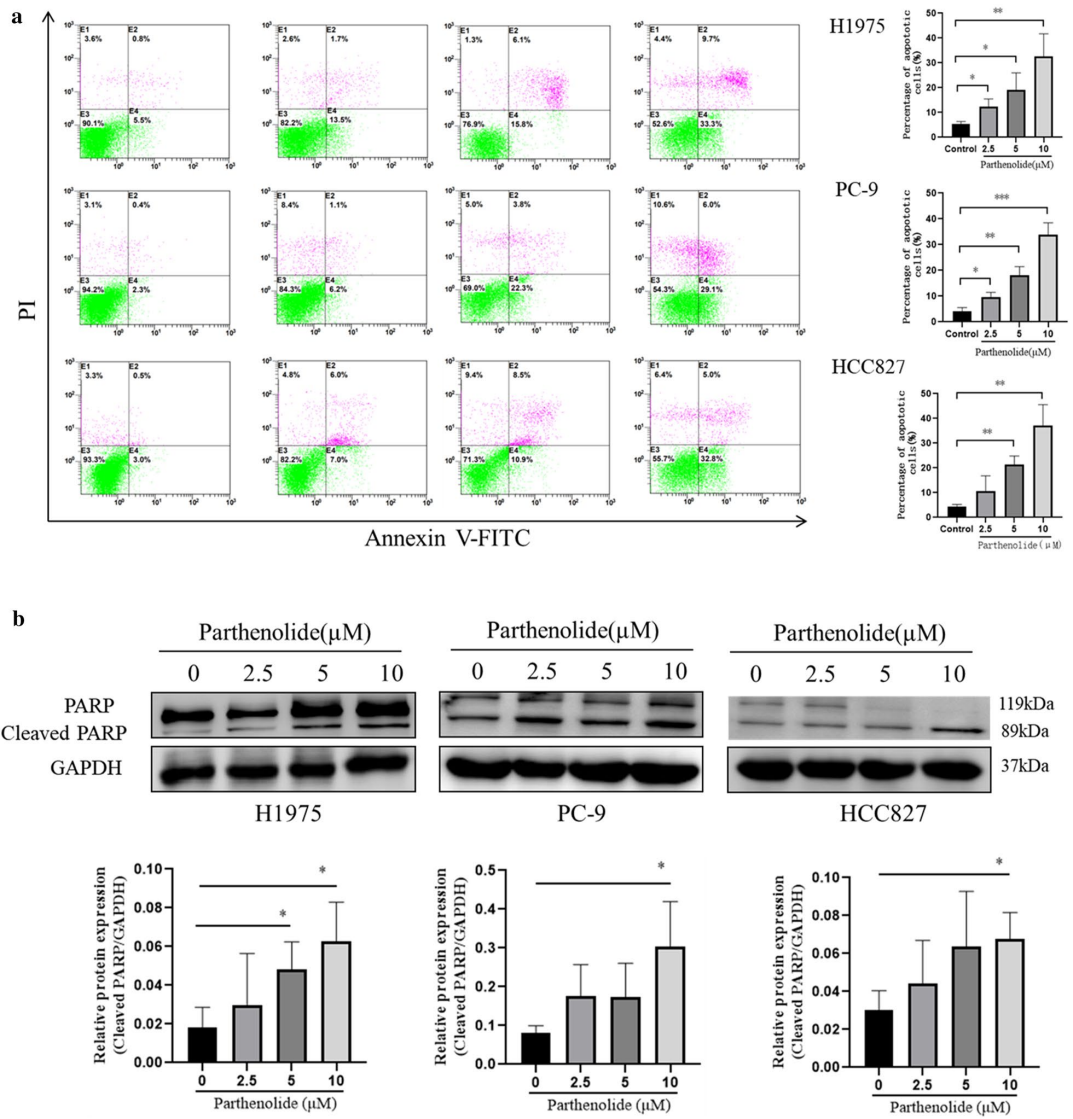
Parthenolide induces apoptosis in NSCLC cells. **a** H1975, PC-9 and HCC827 cells were, respectively, treated with parthenolide at different concentrations (0, 2.5, 5 and 10 µM) for 24 h, apoptosis was analyzed by FITC-Annexin V and PI staining. **b** PARP cleavages were detected by western blotting analysis. The results are representative of three independent experiments. *P < 0.05. **P < 0.01. ***P < 0.001 for comparison between control group and parthenolide-treated group

To investigate whether the induction of apoptosis also contributed to the growth inhibition of mutant EGFR cells mediated by parthenolide, we determined quantify the percentage of apoptosis in EGFR mutated cell lines. As shown in Fig. 2a, parthenolide treatment could significantly induced apoptotic cell death in EGFR mutated cell lines. Further analysis by western blotting showed that PARP (a hallmark of caspase-dependent apoptosis) was cleaved by parthenolide in EGFR mutated cell lines (Fig. 2b). Taken together, these results showed that parthenolide induced apoptosis in EGFR mutated cell lines.

### 3. Parthenolide effectively suppresses EGFR signaling pathway

**Fig. 3:**
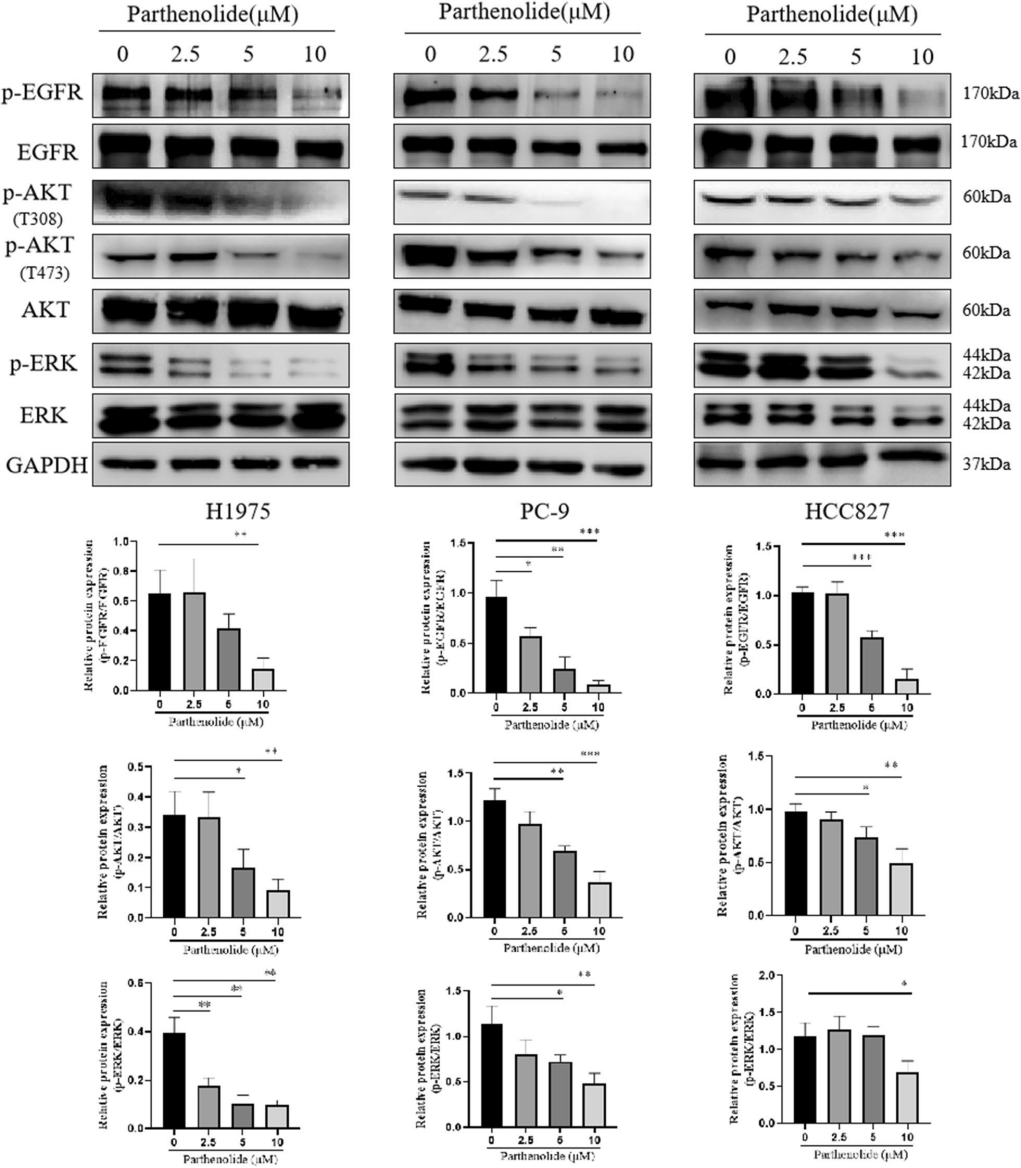
Parthenolide inhibits the EGFR signaling pathway in EGFR mutated NSCLC cells. H1975, PC-9 and HCC827 cells were treated by parthenolide at different concentrations (0, 2.5, 5 and 10 µM) for 24 h. Whole cell lysate was probed to EGFR, phospho EGFR, AKT, phospho AKT (T473 and T308), phospho ERK, ERK and with GAPDH as a loading control. The results are respresentative of three independent experiments. *P < 0.05. **P < 0.01. ***P < 0.001 for comparison between control group and parthenolide-treated group

In order to determine whether parthenolide can inhibit the expression of EGFR in vitro, we observed the effect of parthenolide on EGFR signal pathway in EGFR mutant lung cancer cells. Immunoblotting results showed that parthenolide inhibited the phosphorylation of EGFR in a concentration-dependent manner (Fig. 3). We further evaluated the downstream pathways of EGFR, including ERK and AKT molecules. The phosphorylation of AKT (T473 and T308) and ERK was also inhibited by parthenolide in a concentration-dependent manner, which was consistent with the trend of EGFR phosphorylation. Therefore, our results suggested that parthenolide inhibit phosphorylation of EGFR and its downstream AKT and ERK signaling pathways, which leading to EGFR mutant cell apoptosis and proliferation inhibition.

### 4. Parthenolide effectively inhibits the EGF-induced PI3K/AKT and MEK/ERK signaling pathways

**Fig. 4:**
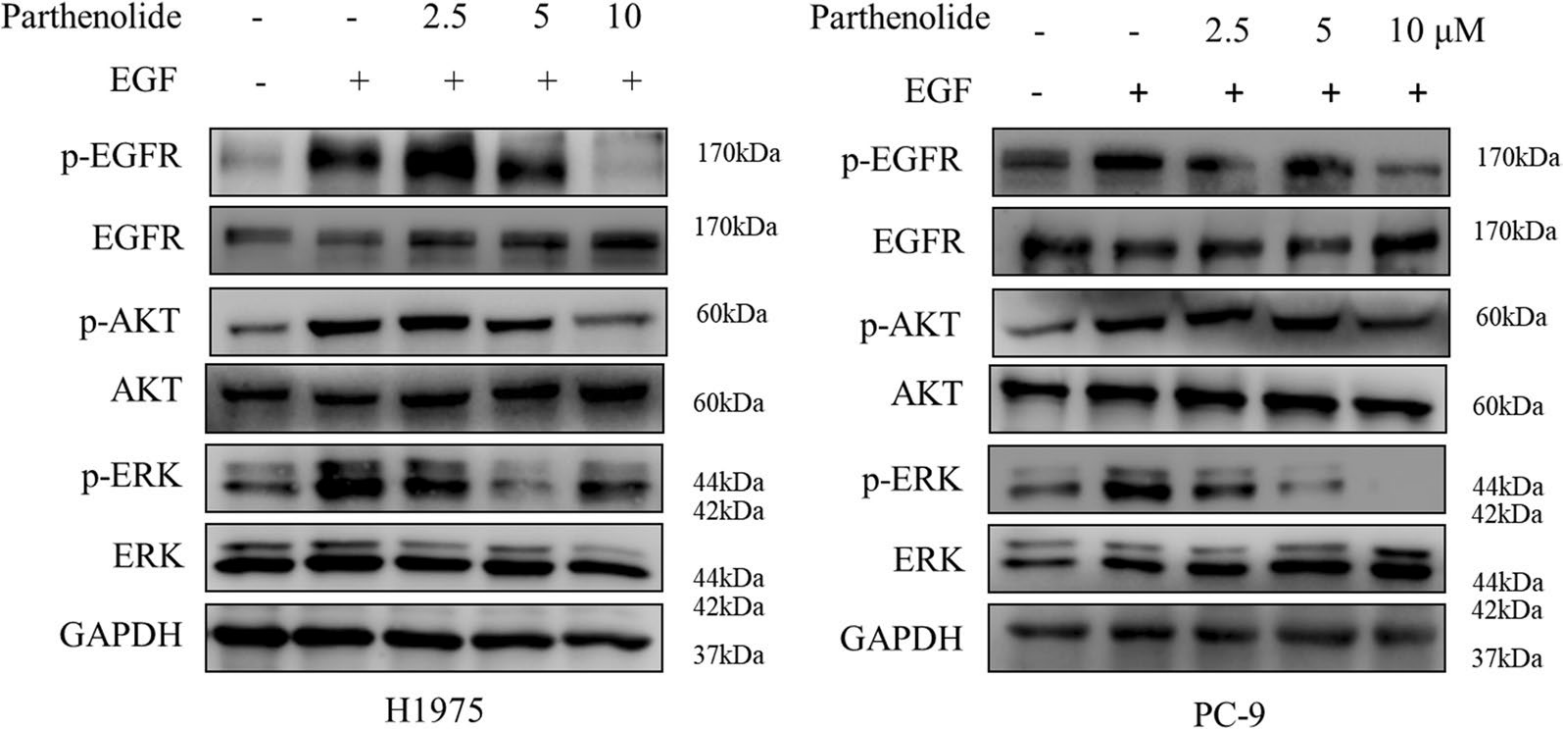
Parthenolide blocks EGF-induced phosphorylation of EGFR, AKT and ERK in EGFR mutated NSCLC cells. H1975 and PC-9 cells were used in EGF stimulation assay in which they were starved in serum-free medium for 16 h and then exposed to different concentrations of parthenolide with or without hEGF treatment. Cells were then collected and subjected to SDS-PAGE, immunoblotted with the indicated antibodies, respectively. GAPDH was used as a loading control in all samples

Human EGF (hEGF) has been reported to bind and activate to EGFR, resulting in activation of PI3K/AKT and MEK/ERK signaling pathways. These two pathways are very important for promoting cell growth and proliferation, and are important downstream signal pathways for EGFR-mediated NSCLC cell proliferation. We speculated that parthenolide may block EGF-induced EGFR activation and its downstream signal transduction. To test this hypothesis, H1975 and PC-9 cell lines were stimulated by EGF. As expected, parthenolide dramatically blocked hEGF-induced phosphorylation of p-EGFR, p-AKT and p-ERK in H1975 and PC-9 cells (Fig. 4). These results suggest that parthenolide can effectively inhibit the phosphorylation of EGFR and the activation of downstream PI3K/AKT and MEK/ERK signal pathways in EGFR mutant lung cancer cells.

### 5. Molecular docking analysis the potential binding of parthenolide to EGFR binding site

**Fig. 5:**
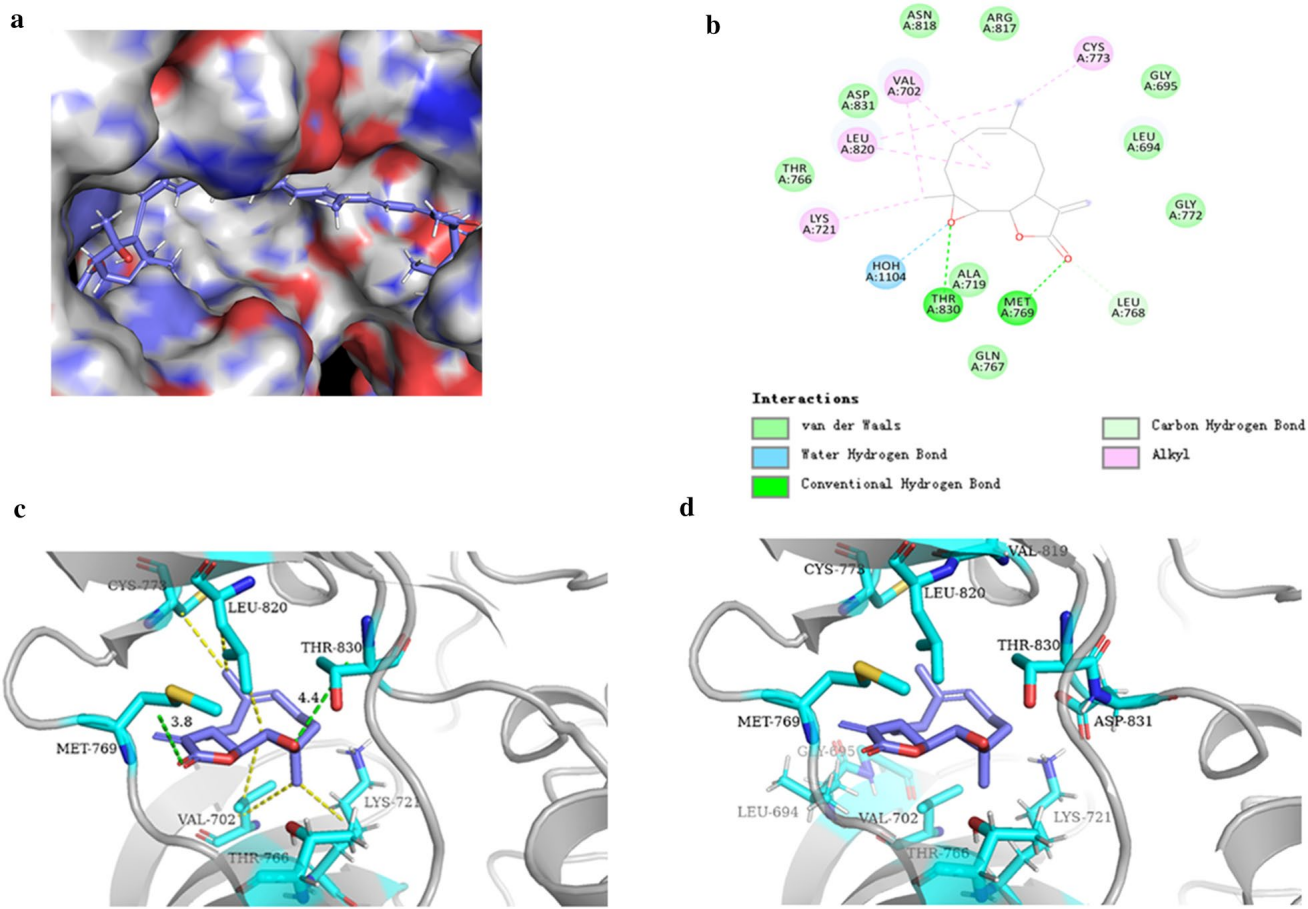
The binding mode of parthenolide between EGFR protein. **a** The hydrophobic surface of EGFR. **b** Schematic representations 2D of the binding interactions between the EGFR active site and parthenlide. **c** Schematic representations 3D of the binding interactions: hydrogen bond interaction between the EGFR active site and parthenlide. **d** Schematic representations 3D of the binding interactions: alkyl between the EGFR active site and parthenlide

We established a model of the interaction between pyrethrolactone and EGFR domain using molecular docking. The results (Fig. 5a) proved that parthenolide could be docked into the kinase domain. The binding model of EGFR and parthenolide revealed that the chain of parthenolide could from strong interactions with THR-830, MET-769, HOH-1104 by theoretical chemistry using the same method. As shown in Fig. 5b, parthenolide can bind to the active region of EGFR through hydrogen bonding and hydrophobic interaction. In addition, the key interactions between battalactone and other domains of EGFR have also been highlighted, including hydrogen bond interaction (green bond) with MET-769, THR-830 (Fig. 5c). As shown in Fig. 5d, parthenolide also exhibited great interactions with LYS-721, LEU-820, CYS-773. These results suggested parthenolide can directly target the active domain of EGFR.

### 6. Network pharmacology analysis of the therapeutic targets of parthenolide

**Fig. 6:**
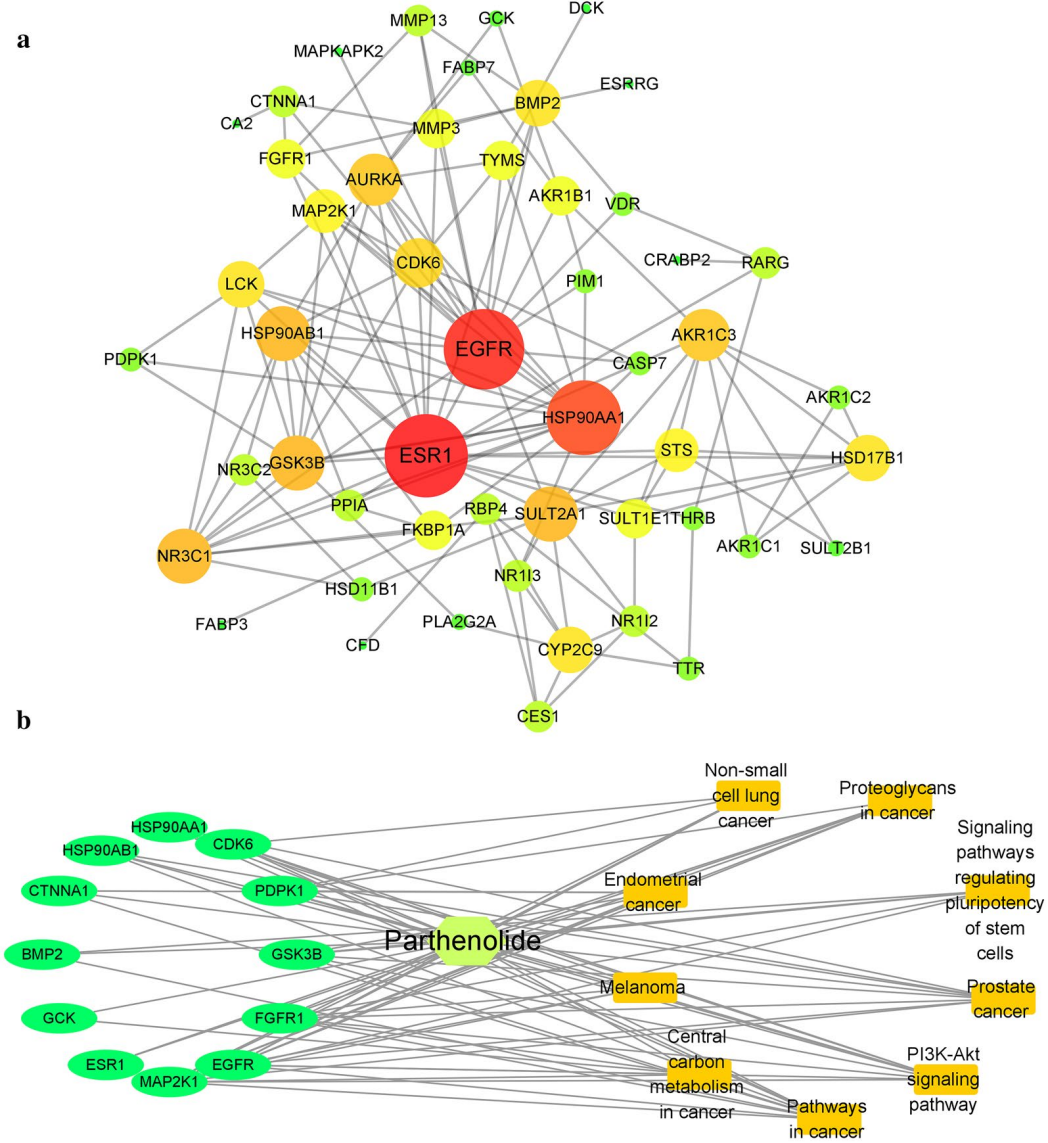
Network pharmacology analysis of the therapeutic targets of parthenolide. **a** Protein-protein interaction of all the candidate protein targets for parthenolide. **b** Compound-gene-pathway-disease-related network diagram of parthenolide

To study the parthenolide target proteins, network pharmacology[25] was employed to predict the interactive proteins and explore possible mechanisms.The results show that parthenolide could exert its anticancer effect through multi-targets and multi-pathways. Through the target protein gene co-expression network (Fig. 6a), it can be seen that the related target protein genes have a certain correlation. Among them, EGFR, ESR1, HSP90AA1 are proteins with a high degree and a high number of directed edges. It can be inferred that parthenolide mainly depends on them to play its role. From the point of view of enriching target protein genes, parthenolide mainly acts on target protein genes such as EGFR. At the same time, these target protein genes are involved in a variety of cancer pathways (Fig. 6b), indicating that parthenolide exhibits potent anticancer activity against NSCLC by targeting EGFR signaling.

### 7. Parthenolide prevents tumor progression in NSCLC xenografts

**Fig. 7:**
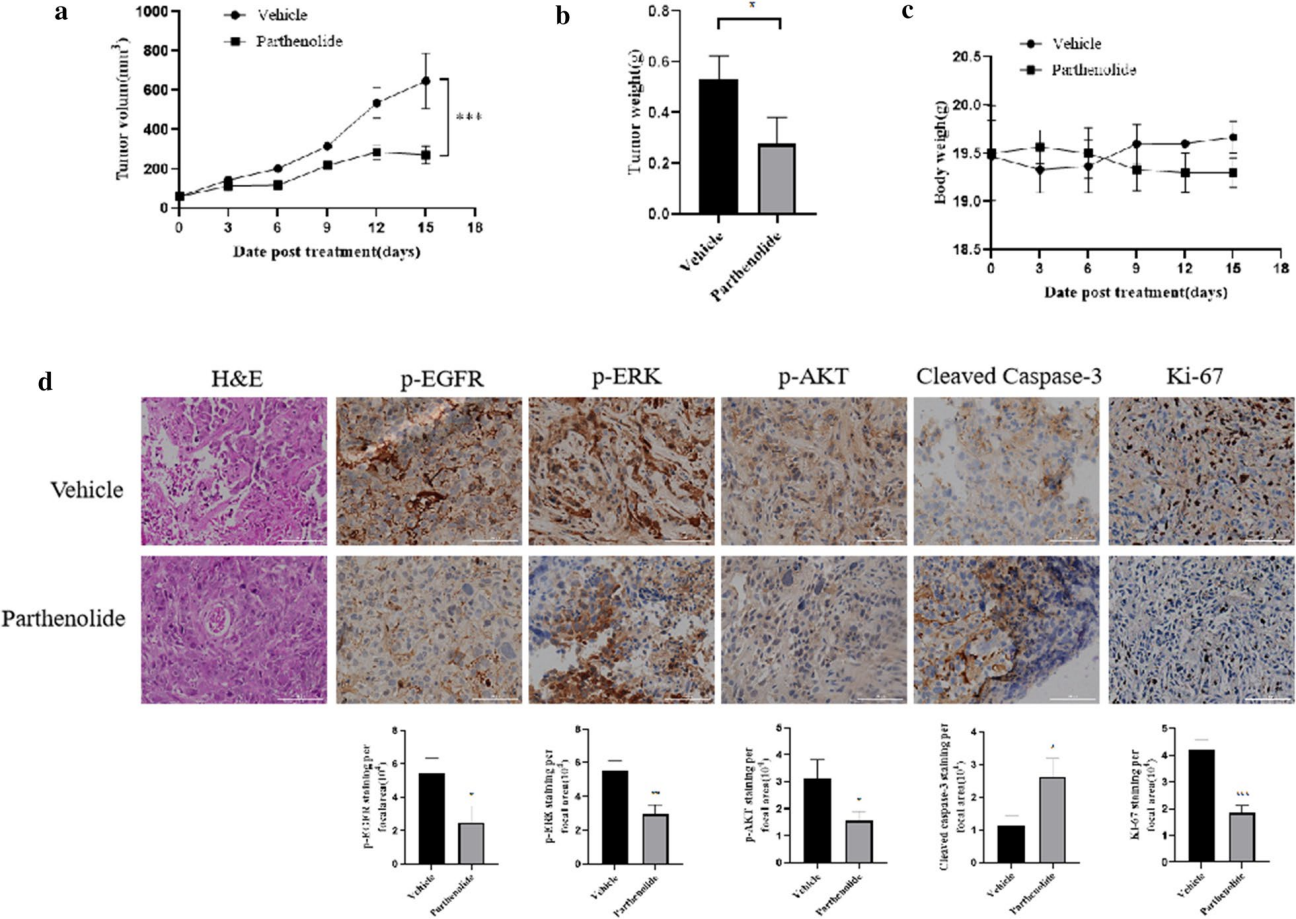
The in vivo effects of parthenolide in NSCLC xenograft therapy model. **a** Nude mice were inoculated with 2.5 million H1975 cells, subcutaneously, into their flanks. **a** Tumor growth curve for H1975 cells xenografts mouse model, when tumors reached ~ 60 mm^3^, mice were randomized into treatment cohorts: vehicle, 20 mg/kg/d parthenolide by ip. Tumor measurements were performed one time per 3 days by calipers, the results were reported as tumor volume (mm^3^) ± SEM. ***P < 0.001, vs. vehicle–treated group. **b** The average tumor weight of control group and the parthenolide-treated tumor at harvest day (day 15). **c** Quantification of body weight for each group. **d** Images represent hematoxylin & eosin (H&E) and immunohistochemical staining for p-EGFR, p-ERK, p-AKT, Ki-67 and cleaved caspase-3 antibody, scale bar, 100 µm. *P < 0.05. **P < 0.01. ***P < 0.001 for comparison vehicle

In order to investigate the anti-tumor effect of parthenolide in vivo, we established a xenograft mouse model by using H1975 NSCLC cells in nudu mice. 7 days after the appearance of relatively small tumors (around 60 mm^3^), parthenolide was formulated for i.p. injection once daily with 20 mg/kg for 15 days, parthenolide did not significantly affect on the body weight of mice (Fig. 7c). Parthenolide significantly inhibited the increase of tumour volume and tumour weight in H1975 xenografts (Fig. 7a, b). Then we further investigate whether parthenolide could suppress EGFR-mediated MEK/ERK and PI3K/AKT cascades *in vivo*, results showed that parthenolide treated mice exhibited prominently increased apoptosis and suppressed cell proliferation as indicated by cleaved caspase-3 and Ki-67 immunostaining, as well as decreased cellularity as measured by HE staining, supporting the observations on the inhibition of tumor growth. Notably, the treatment of parthenolide reduced levels of EGFR, ERK and AKT phosphorylation compared to vehicle-treated mice (Fig. 7d), which consistented with *in vitro* results. Taken together, these results demonstrated that parthenolide is effective in suppressing EGFR mutated lung tumor growth.

## Discussion

Overexpression and mutation of EGFR have been shown to be from normal to early stages of lung adenocarcinoma, the abolition of it is considered as a clinical therapeutic option in NSCLC [5, 26]. Therefore, targeting EGFR is a strategy to inhibit the growth of lung cancer. A number of studies have shown that targeting EGFR can inhibit lung cancer growth including antibodies, small molecule inhibitors, oligonucleotides, peptidomimetics and others [27, 28]. Meanwhile, several natural compounds which is aboundant in the nature have also been found to inhibit EGFR, mainly via effects on downstream signaling pathway in different cancer models [29–31].

As a natural product, parthenolide has been reported to have antitumor activity in many in vitro and in Vivo tumor models [16]. In the current study, we demonstrated that parthenolide treatment decreases the expression of p-EGFR and its downstream molecule AKT and ERK in EGFR mutated lung cancer. It is well known that activation of ERK and AKT has been shown to contribute to tumor growth and progression [32]. Thus, it can be suggested that the cell proliferation inhibition by parthenolide, at least partly, works through the downregulation of EGFR/ERK and EGFR/AKT signaling pathways.

The recognition of molecular targets is an important subject in the study of anticancer effect of drugs. The inhibitory effects of parthenolide on the growth of transplanted tumor mice model: (i) induction of apoptotic cell death of tumor, showing an increase in the level of caleaved caspase-3; (ii) pEGFR, pAkt and pERK were decreased in the tumor samples. Most importantly, we found parthenolide target EGFR through the downregulation of EGFR/ERK and EGFR/AKT signaling pathways.

Network pharmacology is becoming an easily available method combined post-genomic era, systems biology and polypharmacology [33] with blooming amount of data. In addition, the method of network pharmacology is used to study the “complex-protein / gene-disease” approach, which can describe the complexity between biological systems, drugs, and diseases from the perspective of the network, and has a similar overall philosophy to traditional Chinese medicine [25]. Network pharmacology adopts the research strategy of “network target, multi-component”, which replaces the current research method of “one target, one drug” [34, 35]. In recent years, the rapid development of network pharmacology, especially the concept of “network target”, provides a platform for the modernization of Chinese herbal medicine [34]. It provides a new research paradigm for the transformation of Chinese herbal medicine from experience medicine to evidence-based medicine, which will accelerate the discovery of Chinese herbal medicine and improve the existing drug discovery strategy. In this study, we applied network pharmacology to analyze the potential targets and possible mechanisms of anticancer action of parthenolide. Results indicating that parthenolide exhibits potent anticancer activity against NSCLC by targeting EGFR signaling, this result is consistent with molecular docking analysis.

To sum up, we found that parthenolide significantly down-regulated EGFR expression and its downstream signaling pathway in vitro and in vivo, thus inhibiting the growth of NSCLC. These results clearly showed that parthenolide can effectively inhibit the growth of NSCLC lung cancer by down-regulating the expression of ERK and AKT to target EGFR, suggesting that parthenolide could be used as a promising therapeutic candidate for NSCLC patients.

## Data Availability

The data that support the findings of this study are available from the corresponding author upon reasonable request.
